# Impact of complex *NOTCH1 *mutations on survival in paediatric T-cell leukaemia

**DOI:** 10.1186/1471-2407-12-9

**Published:** 2012-01-06

**Authors:** Marcela Braga Mansur, Rocio Hassan, Thayana C Barbosa, Alessandra Splendore, Patricia Y Jotta, José Andrés Yunes, Joseph L Wiemels, Maria S Pombo-de-Oliveira

**Affiliations:** 1 Paediatric Haematology-Oncology Program, Research Centre, Instituto Nacional de Câncer, Rio de Janeiro, RJ, Brazil; 2 Bone Marrow Transplantation Centre (CEMO), Instituto Nacional de Câncer, Rio de Janeiro, RJ, Brazil; 3 Human Genome Studies Center, Instituto de Biociências, Universidade de São Paulo, São Paulo, SP, Brazil; 4 Molecular Biology Laboratory, Centro Infantil Boldrini, Campinas, SP, Brazil; 5 Laboratory for Molecular Epidemiology, Department of Epidemiology & Biostatistics, University of California, San Francisco, CA, USA; 6 Paediatric Haematology-Oncology Program, 6th floor, Research, Center, Instituto Nacional de Câncer-MS, Rua André Cavalcanti, 37, 20231-050 Rio de Janeiro, RJ, Brazil

## Abstract

**Background:**

Molecular alterations occur frequently in T-ALL and the potential impact of those abnormalities on outcome is still controversial. The current study aimed to test whether *NOTCH1 *mutations and additional molecular abnormalities would impact T-ALL outcome in a series of 138 T-ALL paediatric cases.

**Methods:**

T-ALL subtypes, status of *SIL-TAL1 *fusion, ectopic expression of *TLX3*, and mutations in *FBXW7*, *KRAS*, *PTEN *and *NOTCH1 *were assessed as overall survival (OS) and event-free survival (EFS) prognostic factors. OS and EFS were determined using the Kaplan-Meier method and compared using the log-rank test.

**Results:**

The frequencies of mutations were 43.5% for *NOTCH1*, while *FBXW7*, *KRAS *and *PTEN *exhibited frequencies of 19.1%, 9.5% and 9.4%, respectively. In 78.3% of cases, the coexistence of *NOTCH1 *mutations and other molecular alterations was observed. In multivariate analysis no statistical association was revealed between *NOTCH1 *mutations and any other variable analyzed. The mean length of the follow-up was 68.4 months and the OS was 50.7%. *SIL-TAL1 *was identified as an adverse prognostic factor. *NOTCH1 *mutation status was not associated with outcome, while the presence of *NOTCH1 *complex mutations (indels) were associated with a longer overall survival (*p *= 0.031) than point mutations.

**Conclusion:**

*NOTCH1 *mutations alone or in combination with *FBXW7 *did not impact T-ALL prognosis. Nevertheless, complex *NOTCH1 *mutations appear to have a positive impact on OS and the *SIL-TAL1 *fusion was validated as a negative prognostic marker in our series of T-ALL.

## Background

T-cell Acute Lymphoblastic Leukaemia (T-ALL) accounts for ~15% of all childhood ALL cases, and this disease is clinically characterized as a high-risk malignancy with a relapse rate of about 30% [1,2]. T-ALL is also characterized by the occurrence of multiple genetic alterations that result in the transformation of T-cell precursors. Distinct immunophenotypic subsets and somatic genetic alterations have been occasionally correlated with prognosis, but these results could not be consistently replicated by other studies, requiring larger number of cases to confirm associations that could support an improvement in treatment [3-6].

Since the first report addressing the role of *NOTCH1 *mutations in paediatric T-ALL prognosis, several controversial issues have been raised regarding the actual impact of these mutations on prognosis [7]. The *NOTCH1 *gene is expressed in haematopoietic stem cells (HSCs) and controls several steps of T-cell specification and differentiation. This gene was first described in a recurrent t(7;9)(q34;q34) chromosomal translocation rarely found in T-ALL [8], and recently the gain-of-function *NOTCH1 *mutations were reported as a common event in T-ALL patients (~50%) [9]. These mutations mainly involve the heterodimerization (HD) domain, the C-terminal PEST/TAD domain, or both, resulting in up-regulation of Notch1 signalling [9,10]. However, the significance of each type of *NOTCH1 *mutation and especially their impact on disease recurrence remains to be investigated.

Additional molecular markers are not often included in prognostic studies limiting the evaluation of *NOTCH1 *mutations as an independent prognostic factor. Few studies have examined the prognostic role of *NOTCH1 *concurrently with other abnormalities [7]. Genetic lesions targeting multiple cellular pathways including T-lymphoid development, tumour suppression (*FBXW7*) and cell cycle regulation, as well as PI3-kinase/Akt (*PTEN*) and Ras (*KRAS*) signalling appear to be central events in the pathogenesis of T-ALL [11].

Given this evidence, we hypothesized that analysis of *NOTCH1 *in concert with genes functionally related to Notch1 pathway, such as *FBXW7*, *KRAS *and *PTEN*, would provide additional relevant information regarding T-ALL prognosis. We therefore explored associations between the *NOTCH1 *mutations patterns and other somatic alterations in paediatric T-ALL cases in an attempt to better understand the relationship with disease progression and outcome.

## Methods

### Subjects

A series of 138 paediatric T-ALL were selected for this study from 178 cases, from January 2001 to January 2008, based on the availability of biological material for molecular analysis. Subjects were ascertained from four geographical regions assembled by a national network of acute leukaemia studies [12] and the majority of cases were included in a previous publication [6]. The exclusion criteria applied for the analysis was age (12 months) and the diagnosis of T-lymphoblastic lymphoma according to WHO classification [13,14].

Diagnostic samples were obtained prior to any chemotherapy regimen and provided along with demographic and clinical data of each patient. Peripheral blood (PB) samples, typically with high white blood cell (WBC) count, were send along with BM aspirates. The presence or absence of blast cells in the BM aspirate and PB was reviewed before molecular analysis. To leukaemia diagnosis a threshold of >20% of blast cells in the BM was used as lower limit for analysis. In the PB, a clinically high WBC count or a blast percent ≥50% were considered suitable for our analysis.

### Ethical aspects

The Ethical and Scientific Committees of the Instituto Nacional de Câncer, Rio de Janeiro, Brazil approved the study (CEP-INCA#107/06) in accordance with the Declaration of Helsinki ethical standards.

### Demographic and clinical data

Characteristics of patients obtained at diagnosis were age, gender, mediastinum involvement and elevated WBC counts. The participants' ethnicities were categorized according to the skin colour, as Whites and non-Whites. There were 60 (43.5%) patients self reported as Whites and 78 (56.5%) as non-Whites. In the non-White category blacks and all mixed-populations were included, according to criteria described elsewhere [15]. The age range was ≥1-18 years-old, which was divided in two groups for data analysis as < 10 and 10-18 years-old. Eighty six cases (62.3%) were from the Northeast region of Brazil, 34 (24.6%) from the Southeast, 15 (10.9%) cases from Central plateau; and 3 (2.2%) from the South region.

One-hundred and one patients were treated according to Brazilian Group for Treatment of Childhood Leukaemia (GBTLI-99), while 37 were treated according to the ALL-Berlin-Frankfurt-Munster (BFM) protocols backbone strategies, as described elsewhere [16,17].

### Immunological characterization

In all cases, the diagnosis was established according to criteria previously defined [13,14]. After mononuclear cell separation using Ficoll-Hypaque-Hystopaque^®^, immunophenotype was performed in blast cells by flow cytometry using a defined panel of monoclonal antibodies, as previously described [6] and/or by immunohistochemistry methods [13,14]. Immunological classification of T-ALL was based on criteria previously published by the European Group for the Immunological Characterization of Leukaemias [18].

### Nucleic acids extraction and cDNA synthesis

Genomic DNA was extracted from BM aspirate by one of the following methods: the QIAamp^® ^DNA Blood Mini Kit (Qiagen GmbH, Hilden, Germany) (n = 78), ethanol precipitation from phenol phase after RNA isolation (n = 36), or the salting out method (n = 24).

For RT-PCRs assays, the total RNA was prepared using the TRIzol reagent kit (Invitrogen, Carlsbad, California, USA) according to the manufacturer's instructions (n = 129). cDNA was generated by reverse transcription of 2 μg of total RNA using First-Strand cDNA Synthesis Kit™ (Amersham Biosciences UK Limited, Little Chalfont, UK), and stored at −20°C. The integrity of cDNA was examined by amplifying a fragment of the *GAPDH *constitutive gene, according to reported conditions [6].

### *NOTCH1 *mutations

*NOTCH1 *mutations were analyzed by screening of the heterodimerization (HD) and polypeptide enriched in proline, glutamate, serine and threonine (PEST) domains according to procedures described previously [9]. In general, PEST and TAD domains are both denominated PEST domain. In summary, the N-terminal region of the HD domain of *NOTCH1*, encoded by exon 26, was divided in two amplicons. The amplification of exon 27, encoding the C terminal region of *NOTCH1 *HD domain, resulted in one amplicon; and sequences of exon 34 encoding the PEST domain and the contiguous N-region containing the TAD domain were amplified as three amplicons. All six amplicons were sequenced on both strands. Mutations were classified into two groups: complex and point mutations. Complex mutations were defined as insertions and/or deletions (also called as indels) in the gene sequence, and point mutations as single base changes. A case was considered to harbour complex mutation when an indel was detected alone or concomitant with point mutations. Classification in missense or nonsense mutations was in accordance with classical mutation definition.

### *FBXW7 *mutations

To evaluate the mutational status of *FBXW7*, we screened the exons 9 and 10, previously reported to be the most frequently mutated regions [19,20]. We performed PCR reactions for each exon with specific primers. PCR products were purified and then directly sequenced. All procedures for *FBXW7 *screening were described elsewhere [20].

### *SIL-TAL1 and TLX3 *assays

The *SIL-TAL1 *fusion and *TLX3 *(also known as *HOX11L2*) presence were investigated according to previously published methods [21,22]. PCR products of both RT-PCR assays were separated by 1.5% agarose gel electrophoresis, stained with ethidium bromide.

### *KRAS *mutations

To study the mutations in codons 12 and 13 of the *KRAS *gene were carried out separate PCR reactions for each codon, using 100 ng of genomic DNA. PCR products from codon 12 were digested with restriction enzyme using NE Buffer 2, 100 μg/ml BSA and 10U of *BstNI *(Biolabs, New England, MA), and incubated overnight at 60°C. The PCR products of codon 13 were digested with 2U of *PflMI *for 6 h at 37°C. The digested PCR fragments were visualized on a 3% agarose gel, as previously described [23]. All the mutations found with restriction fragment length polymorphism- RFLP assay were confirmed by direct sequencing.

### *PTEN *mutations

*PTEN *sequences of exons 1 and 7 were amplified by PCR. Conformational analysis was performed by heteroduplex assays, followed by direct DNA sequencing to confirm abnormal patterns, as described elsewhere [24]. PCR products were sequenced on both strands using the BigDye kit (Applied Biosystems).

### Statistical analysis

Univariate *p *values were calculated using Pearson's chi-square test. Mann-Whitney test was used to test median differences of continuous variables. Two-sided *p *values with a significance limit of 0.05 were used throughout the study. The probability of overall survival (OS) was determined using the Kaplan-Meier method in months from the diagnosis to outcome (death, alive or last follow-up). Patients lost to follow-up were censored at their date of last known contact. Event-free survival (EFS) was the interval (in months) from diagnosis to progression at any time, relapse from complete response, or initiation of new, previously unplanned treatment or to the last follow-up in the patients with treatment success. The differences between T-ALL survival distributions were compared by the log-rank test. The multivariate Cox proportional hazard regression method was used to determine the independent prognostic factors influencing OS and EFS. Multivariate Cox analysis was performed with variables associated with a *p *≦ 0.1 in univariate analysis such as the type of *NOTCH1 *mutation (point vs. complex mutations), *SIL-TAL1 *fusion and induction responses. SPSS (Statistical Product and Services Solutions, version 18.0, SPSS Inc, Chicago, IL, USA) software was used for data analyses.

## Results

The main demographic, clinical, and molecular characteristics of T-ALL cases according to *NOTCH1 *status are shown in Table 1. The median age of the total series of cases was 8 years (1-18 years), and a predominance of males was observed, with a 2.6:1 male/female ratio. Immunophenotyping analyses by flow cytometry were available for 130 cases, and in 8 cases the T-cell diagnosis were confirmed by immunohistochemistry with CD45^+^/CD3^+^. Regarding T-cell maturation, we observed a predominance of T-IV subtype (38.0%). Overall, 60/138 patients (43.5%) presented with *NOTCH1 *mutations; additional molecular alterations observed were *FBXW7 *mutations (21/110; 19.1%), *SIL-TAL1 *fusion (37/129; 28.7%), *TLX3 *ectopic expression (10/119; 8.4%), *KRAS *(10/105; 9.5%) and *PTEN *mutations (9/96; 9.4%). In 78.3% of cases, the coexistence of *NOTCH1 *mutations and other molecular alterations was observed, including *FBXW7 *(n = 13), *SIL-TAL1 *(n = 19), *TLX3 *(n = 6), *PTEN *(n = 5), and *KRAS *(n = 4) mutational. However, no statistical association was disclosed between *NOTCH1 *mutations status and any other variable analyzed.

**Table 1:** Clinical, demographic and laboratorial features of T-ALL patients according to *NOTCH1 *status, 2001-2008

Variable	n (%)	*NOTCH1 *mut n (%)	*NOTCH1 *wt n (%)	*p value*
Age (Years)				
< 10	71 (51.4)	28 (46.7)	43 (55.1)	*0.324*
10-18	67 (48.6)	32 (53.3)	35 (44.9)	
Gender				
Male	100 (72.5)	46 (76.7)	54 (69.2)	*0. 332*
Female	38 (27.5)	14 (23.3)	24 (30.8)	
WBC (×10^9^/L)				
<50	63 (45.7)	29 (48.3)	34 (43.6)	*0.579*
≥50	75 (54.3)	31 (51.7)	44 (56.4)	
Mediastinal mass^a^				
Yes	58 (44.6)	24 (42.9)	34 (45.9)	*0.726*
No	72 (55.4)	32 (57.1)	40 (54.1)	
Skin colour				
White	60 (43.5)	21 (35.0)	39 (50.0)	*0.078*
Non-white	78 (56.5)	39 (65.0)	39 (50.0)	
T-ALL subtypes^b^				
T-I	18 (13.8)	9 (15.5)	9 (12.5)	*0.343*
T-II	29 (22.3)	9 (15.5)	20 (27.8)	
T-III	34 (26.2)	18 (31.0)	16 (22.2)	
T-IV	49 (37.7)	22 (37.9)	27 (37.5)	
CD10 status^c^				
positive	36 (31.0)	16 (30.2)	20 (31.7)	*0.857*
negative	80 (69.0)	37 (69.8)	43 (68.3)	
Molecular Studies				
*SIL-TAL1 *^pos^	37 (28.7)	19 (32.8)	18 (25.4)	*0.355*
*HOX11L2 *^pos^	10 (8.4)	6 (11.1)	4 (6.2)	*0.332*
*FBXW7 *^mut^	21 (19.1)	13 (24.1)	8 (14.3)	*0.192*
*KRAS *^mut^	10 (9.5)	4 (7.7)	6 (11.3)	*0.527*
*PTEN *^mut^	9 (9.4)	5 (10.9)	4 (8.0)	*0.630*

Total	138 (100)	60 (100)	78 (100)	

*n *number of cases, *mut *mutated, *wt *wild-type, *WBC *white blood cells count at diagnosis. ^a ^8 cases without information about mediastinal mass, being 4 with *NOTCH1 *mutation and 4 wild-type; ^b ^classification according to EGIL criteria, flow cytometry was not performed in 8 cases, 2 presented *NOTCH1 *mutation and 6 wild-type; ^c ^there were 22 cases without CD10 status evaluated, 7 with *NOTCH1 *mutation and 15 wild-type; pos, positive; neg, negative

Considering that *NOTCH1 *and *FBXW7 *belong to the same signalling pathway, patients with mutations in both genes (*NOTCH1 *and/or *FBXW7*) were analyzed together as a *NOTCH1-FBXW7 *mutated group. In total, 62 patients (56.4%) presented with *NOTCH1-FBXW7 *mutations. Patients with *FBXW7*, *KRAS *or *PTEN *mutations shared other molecular alterations along with *NOTCH1 *mutations, such as *FBXW7 *mutation and *SIL/TAL1 *(n = 5), *FBXW7 *mutation and *TLX3 *(n = 1), *FBXW7 *and *KRAS *mutations (n = 2), *FBXW7 *and *PTEN *mutations (n = 1). We also observed *KRAS *mutation and *SIL/TAL1 *(n = 1), *KRAS *mutation and *TLX3 *(n = 2), *PTEN *mutation and *SIL-TAL1 *(n = 1), *PTEN *and *KRAS *(n = 1), and one case exhibited *PTEN*, *KRAS *and *SIL-TAL1 *alterations.

The complete descriptions of *NOTCH1 *mutations sequences in HD and PEST domains are shown in Additional file 1: Table S1. Single nucleotide polymorphism (SNP) already described, recurrent mutations as well as previously unreported mutations were observed. Thirty-eight new mutations were found in the HD domains and 15 in PEST domain, representing 63.1% (53/84) of all mutations found. Among these new mutations there were no SNPs, since we have checked and excluded any SNP possibility through NCBI SNP database and 1000 genomes data. The *NOTCH1 *SNPs found in this work have been excluded from all subsequent mutation analyses; and they were mentioned in Additional file 1: Table S1 only for descriptive purposes.

The main demographic and clinical features of T-ALL patients with different types of *NOTCH1 *mutations are summarized in Table 2. Mutations were not randomly distributed, with a predominance of point mutations in the HD domain, and complex mutations in the PEST domain (*p *= 0.014). Also, nonsense mutations were more frequent in the PEST domain and missense mutations in the HD domain (*p *< 0.0001). Seven cases presented with mutations in both HD and PEST domains, with a predominance of complex mutations (22.2%). No significant statistical differences were observed with respect to age groups, gender and T-ALL subtypes, in respect to any type of *NOTCH1 *mutation. High WBC was observed in patients harbouring complex mutations, while patients with point mutations exhibited lower WBC (*p *= 0.035). Point mutations were more frequent in non-white patients (*p *= 0.053); where a higher frequency of mutations in the HD domain was observed (89.7% *versus *only 10.3% in PEST domain, *p *= 0.028). Missense mutations were more frequent in CD10+ cases (*p *= 0.042). Eleven out of 14 nonsense mutations were caused by complex mutations, the other three by point mutations. Thirty out of 46 missense mutations were caused by point mutations and the remaining 16 by complex mutations (*p *= 0.004, data not shown).

**Table 2:** Main demographic and clinical features of T-ALL patients according to type and classification of *NOTCH1 *mutations

*Variables*	Point Mutations^a ^(%)	Complex Mutations n (%)	*p value*	Missense n (%)	Nonsense n (%)	*p value*
Age (Years)						
< 10	14 (42.4)	14 (51.9)	*0.466*	21 (45.7)	7 (50.0)	*0.775*
10-18	19 (57.6)	13 (48.1)		25 (54.3)	7 (50.0)	
Gender						
Male	26 (78.8)	20 (74.1)	*0.668*	34 (73.9)	12 (85.7)	*0.361*
Female	7 (21.2)	7 (25.9)		12 (26.1)	2 (14.3)	
WBC (x10^9^/L)						
<50	20 (60.6)	9 (33.3)	*0.035*	23 (50.0)	6 (42.9)	*0.640*
≥50	13 (39.4)	18 (66.7)		23 (50.0)	8 (57.1)	
Skin colour						
White	8 (24.2)	13 (48.1)	*0.053*	15 (32.6)	6 (42.9)	*0.481*
Non-white	25 (75.8)	14 (51.9)		31 (67.4)	8 (57.1)	
T-ALL subtypes^b^						
T-I	4 (12.9)	5 (18.5)	*0.825*	8 (17.8)	1 (7.6)	*0.589*
T-II	4 (12.9)	5 (18.5)		6 (13.3)	3 (23.1)	
T-III	10 (32.3)	8 (29.6)		15 (33.3)	3 (23.1)	
T-IV	13 (41.9)	9 (33.3)		16 (35.6)	6 (46.2)	
CD10 status^c^						
Positive	11 (39.3)	5 (20.0)	*0.127*	15 (37.5)	1 (7.7)	*0.042*
Negative	17 (60.7)	20 (80.0)		25 (62.5)	12 (92.3)	
NOTCH1 mutated domain						
HD	28 (84.8)	14 (51.9)	*0.014*	38 (82.6)	4 (28.6)	*<0.0001*
PEST/TAD	4 (12.1)	7 (25.9)		4 (8.7)	7 (50.0)	
Both domains	1 (3.0)	6 (22.2)		4 (8.7)	3 (21.4)	
*FBXW7*status^d^						
Mutated	6 (20.0)	7 (29.2)	*0.434*	11 (27.5)	2 (14.3)	*0.320*
WT	24 (80.0)	17 (70.8)		29 (72.5)	12 (85.7)	

Total	33 (100)	27 (100)		46 (100)	14 (100)	

^a ^insertions and/or deletions; WBC, white blood cells count; ^b ^classification according to EGIL criteria, flow cytometry was not performed in 2 cases with mutations, both were classified as point/missense; ^c ^CD10 status was not available in 7 cases, being 5 with point mutations and 2 complex; and 6 classified as missense and 1 as nonsense; ^d ^*FBXW7 *mutational status was not performed in 28 cases out of 138, 22 of 28 were WT, 3 presented point mutations and 3 complex mutations, all 6 mutations were classified as missense

**Figure 1:**
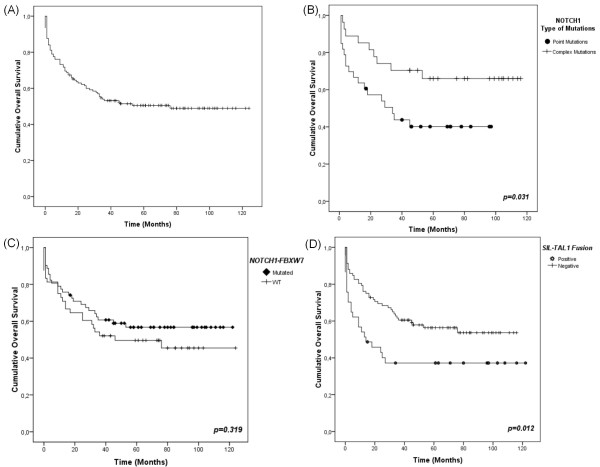
**Kaplan-Meier overall survival curves for T-ALL patients**. (A) Overall survival (OS) of T-ALL patients. In this analysis were included all 138 patients with T-ALL. (B) *NOTCH1 *type of mutation (point mutations vs. complex mutations) OS curve. For the construction of this curve were included all 60 patients with *NOTCH1 *mutations, being 33 patients harbouring point mutations and 27 complex mutations. (C) OS curve for *NOTCH1*-*FBXW7 *status. In this Kaplan-Meier OS curve were analyzed 110 T-ALL patients with *NOTCH1*-*FBXW7 *status determined, 62 with *NOTCH1*-*FBXW7 *mutations and 48 displaying WT profile. (D) OS according to the presence or absence of *SIL-TAL1 *fusion gene. Symbols represent censored cases. The *SIL-TAL1 *fusion OS analysis included 129 T-ALL cases, being 37 *SIL-TAL1+ *and 92 *SIL-TAL1-*.

Overall survival of the 138 cases was 50.7% (Figures 1A). Mean length of the follow-up was 68.4 months (95% CI 58.9-77.8 months). For 68 patients who evolved to death, the follow-up was 0-76 months (median 9), with 9 deaths occurring in the first 3 days after diagnosis and 57.4% of deaths (39 patients) occurring in the first 12 months of treatment. For 70 patients alive, the follow-up was 15-124 months (median 73.5), with 75% of patients with 94.5 months follow-up. No differences in OS were observed in patients treated with GBTLI or BFM-based protocols (52.5% vs. 45.9%, respectively; *p *= 0.639).

The results of the OS univariate analyses of T-ALL cases considering variables such as age, WBC at onset of disease, T-ALL cellular subtypes, CD10 expression, skin colour, response to induction therapy, mutational status of *NOTCH1*, *FBXW7*, *NOTCH1-FBXW7 *group, *KRAS *and *PTEN*, presence of *SIL-TAL1 *fusion, and ectopic expression of *TLX3 *are shown in Table 3, Figure 1 and Additional file 2: Figure S1.No statistically significant impact in OS was found for the following variables: age, WBC, T-ALL subtypes, CD10 expression, skin colour, mutational status of *FBXW7*, *KRAS *and *PTEN*, and ectopic expression of *TLX3*.

**Table 3:** Univariate analysis of overall (OS) and event-free survival (EFS) in T-ALL patients

Variables	OS^a ^(95% CI)	*p*^b^	HR^c ^(Lower-Upper)	EFS^a ^(95% CI)	*p*^b^	HR^c ^(Lower-Upper)
Age (Years)						
< 10	45.1 (48.9-74.5)	*0.275*	0.77 (0.48-1.24)	28.8 (30.8-57.8)	*0.044*	0.58 (0.34-1.00)
10-18	56.7 (60.7-88.0)			51.2 (51.9-85.8)		
WBC (x10^9^/L)						
<50	47.6 (51.6-79.4)	*0.606*	0.88 (0.55-1.42)	41.3 (40.9-73.3)	*0.859*	1.05 (0.63-1.76)
≥50	53.3 (57.1-82.5)			36.7 (39.2-68.5)		
EGIL						
T-I	33.3 (28.8-77.2)	*0.411*	0.93 (0.74-1.18)	31.3 (26.9-75.9)	*0.954*	0.98 (0.77-1.24)
T-II	51.7 (48.2-88.7)			36.4 (31.5-75.5)		
T-III	61.8 (56.1-90.7)			45.0 (32.9-78.6)		
T-IV	51.0 (44.0-70.9)			36.7 (30.2-60.9)		
CD10						
Positive	52.8 (50.6-86.9)	*0.696*	1.12 (0.63-1.97)	48.1 (42.6-83.6)	*0.097*	1.67 (0.89-3.08)
Negative	50.0 (54.1-79.4)			28.8 (31.6-59.5)		
Skin colour						
White	55.0 (54.5-81.5)	*0.451*	1.20 (0.74-1.96)	33.3 (32.1-61.5)	*0.331*	0.78 (0.46-1.30)
Non-white	47.4 (52.8-77.8)			43.4 (45.9-75.9)		
*NOTCH1*						
Mutated	53.3 (54.6-80.8)	*0.479*	1.19 (0.73-1.93)	34.2 (30.5-60.9)	*0.382*	0.79 (0.47-1.34)
WT	48.7 (52.7-78.2)			42.1 (45.5-74.2)		
Type of mutation						
Point	42.4 (32.4-61.9)	*0.031*	0.43 (0.19-0.95)	30.4 (19.1-51.4)	*0.289*	0.65 (0.28-1.47)
Complex	66.7 (65.6-100.9)			40.0 (31.4-79.4)		
*FBXW7*						
Mutated	61.9 (54.8-102.3)	*0.512*	1.28 (0.60-2.73)	58.3 (38.9-104.9)	*0.586*	1.29 (0.51-3.31)
WT	51.7 (59.2-82.3)			41.4 (47.2-74.7)		
*NOTCH1*-*FBXW7*						
Mutated	58.1 (62.5-89.6)	*0.319*	1.32 (0.76-2.28)	40.5 (37.6-72.3)	*0.286*	0.71 (0.37-1.34)
WT	47.9 (50.7-82.3)			48.5 (51.1-87.7)		
*SIL-TAL1*						
Positive	37.8 (32.1-68.3)	*0.012*	0.53 (0.32-0.88)	18.2 (11.9-48.7)	*0.004*	0.46 (0.26-0.81)
Negative	56.5 (60.9-81.9)			43.8 (46.4-71.1)		
*HOX11L2*						
Expressed	40.0 (13.4-44.6)	*0.469*	0.74 (0.32-1.71)	50.0 (14.3-50.2)	*0.806*	1.14 (0.41-3.16)
Absent	48.6 (54.1-75.2)			35.6 (39.3-63.6)		
*KRAS*						
Mutated	60.0 (32.7-78.1)	*0.585*	1.32 (0.48-3.67)	57.1 (21.4-79.7)	*0.584*	1.38 (0.43-4.46)
WT	49.5 (55.7-77.9)			36.9 (41.5-66.9)		
*PTEN*						
Mutated	44.4 (15.1-56.1)	*0.359*	0.65 (0.26-1.66)	30.8 (34.6-66.1)	*0.693*	0.31 (0.53-1.51)
WT	57.5 (63.5-86.6)			42.0 (41.0-71.8)		
Induction Response^d^						
Yes	68.4 (76.2-106.9)	*<0.001*	3.45 (1.74-6.85)	70.3 (75.5-107.7)	*<0.001*	3.54 (1.71-7.32)
No	33.3 (25.9-55.9)			32.4 (23.4-48.6)		

^a ^Survival in percent; ^b ^Mantel-Cox (log-rank) test; ^c ^last category used as reference; ^d ^evaluated in day 30-33 of treatment; *HR *hazard risk, *WT *wild-type, *WBC *white blood cells count

Test of equality of survival distribution for the differences of *NOTCH1 *status (mutated vs. wild-type) showed no statistical significant results (53.3% vs. 48.7%; *p *= 0.479). On the other hand, carriers of *NOTCH1 *complex mutations (indels) exhibited a favourable OS when compared with carriers of point mutations (66.7% vs. 42.4%; *p *= 0.031; HR 2.33, CI95% 1.05-5.18) (Figures 1B), *NOTCH1 *wild-type patients showed an intermediate OS rate (48.7%). OS differences between complex and point *NOTCH1 *mutations were more marked in the patients treated with the BFM protocols backbone strategies (27.3% vs. 62.5%; *p *= 0.041). For *NOTCH1*-*FBXW7 *combined mutations no impact on OS was observed (58.1% vs. 47.9%), mutated in *NOTCH1-FBXW7 *vs. WT; *p *= 0.319 showed in Figure 1C.

The presence of *SIL-TAL1 *fusion was predictive of a worse outcome (OS 37.8% vs. 56.5%; *p *= 0.012; HR 1.89, CI95% 1.13-3.17) (Figure 1D). Multivariate Cox analysis showed that the type of *NOTCH1 *mutations (point vs. complex mutations) was not an independent prognostic factor (*p *= 0.769), compared with *SIL-TAL1 *fusion (*p *= 0.04; HR 2.87; 95% CI 1.39-5.92) and induction response (*p *= 0.10; HR 2.69; 95% CI 1.27-5.71), even when adjusted by treatment protocol.

In 80 cases, the initial response to induction phase (day 30-33) was recorded, with 52.5% of cases exhibiting induction failure, independent on protocol allocation. Of the 38 cases that achieved clinical remission, 26 (72.5%) maintained the initial response and 12 (27.5%) relapsed during maintenance therapy and died. Of 42 cases with induction failure 11 (26.2%) evolved to death and the remaining 31 were submitted to re-induction therapy (14 with secondary remission).

Induction therapy response was significantly associated with a longer overall survival, both in univariate and multivariate analyses (*p *< 0.0001; HR 2.86; 95% CI 1.38-5.94) as shown in Table 3. However, no significant association was found between any of the molecular alterations and induction response (data not shown).

Ninety-five patients had sufficient clinical data for conducting EFS analysis (9 cases that evolved to death previously to treatment due to severity of disease were excluded of this analysis). All variables evaluated in the OS analysis were also included in EFS investigations. Results of EFS analysis are described in Table 3.

**Figure 2:**
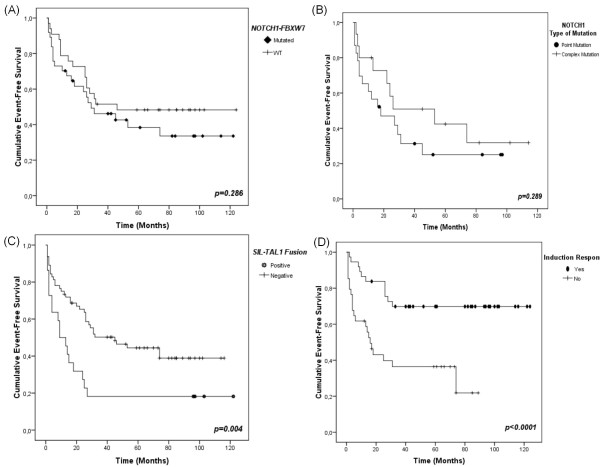
**Kaplan-Meier event-free survival curves (EFS) for T-ALL patients**. For the EFS endpoint we performed survival analysis in 95 out of 138 T-ALL patients. (A) EFS curve for patients harbouring *NOTCH1*-*FBXW7 *mutations vs. WT cases. In the construction of this EFS curve were included 70 patients, 37 with *NOTCH1*-*FBXW7 *mutations and 33 WT. (B) EFS according to the type of *NOTCH1 *mutations (complex vs. point). This curve is corresponding to analysis of 38 patients with EFS and *NOTCH1 *type of mutations available data. Twenty-three patients presented point mutations and 15 complex mutations. (C) *SIL-TAL1 *fusion negative impact on EFS. Eighty-six patients were analyzed for *SIL-TAL1 *prognostic role in EFS, 22 *SIL-TAL1+ *and 64 *SIL-TAL1-*. (D) EFS of patients that respond to induction compared to those without response. Symbols represent censored cases. For the construction of this EFS curve were analyzed 71 patients, being 37 with induction response and 34 with no response.

EFS exhibited no difference according to *NOTCH1*-*FBXW7 *status (40.5% Mutated, 48.5% WT, *p *= 0.286) (Figure 2A). For patients bearing complex *NOTCH1 *mutations the EFS was better than for those presenting point mutations (30.4%, 40% respectively, *p *= 0.289), albeit not statistically significant (Figure 2B). EFS was significantly worse in patients bearing *SIL-TAL1 *(18.2% vs. 43.8%; *p *= 0.004) (Figure 2C), and also in those patients with no response to induction therapy (32.4% vs. 70.3%, *p *< 0.0001) (Figure 2D). Multivariate Cox analysis identified only *SIL-TAL1 *(HR 3.12; 95% CI 1.48-6.57) and induction failure (HR 3.07; 95% CI 1.44-6.55) as independent negative prognostic factors.

## Discussion

In a previous analysis, we compared the presence of CD1a^+ve ^phenotype, *SIL-TAL1 *status, *TLX3 *abnormal expression and the clinical features on the impact in the prognosis of T-ALL in series of Brazilian children and young adults [6]; whereas the presence of *SIL-TAL1 *had a poor outcome, particularly in younger children, either ectopic *TLX3 *or immunophenotyping were not predictive factors for outcome corroborating with data described by the Dutch Childhood Oncology Group [25]. Albeit, molecular alterations such as *SIL-TAL1*, *TLX3*, and *NOTCH1 *mutations have been reported as T-ALL prognostic factors, due to scarce studies world-wide, most of them have inconsistent results [3,10,25,26].

Investigation of *NOTCH1 *mutations and their relationships with T-cell specific markers is still relevant because of its importance as a potential therapeutic target and prognostic marker [27]. In this context, the current study is one of the largest homogenous series of T-ALL childhood patients incorporating *NOTCH1 *mutation screening. Overall the *NOTCH1 *mutations were not correlated with variables considered as prognostic factors such as, age range, T-cell subtypes or other molecular aberrations. However, new interesting information concerning the association between WBC count and type of *NOTCH1 *mutation was observed (*p *= 0.035). This finding cannot be compared with other data, since there is no similar analysis reported on the literature.

The frequency of *NOTCH1 *mutation in our series of patients was 43.5%, which is consistent with the results of previously reported clinical series, that showed mutation frequencies between 31 and 62%, depending on the series size and age groups [3,9,25,28,29]. The *NOTCH1 *mutation screening found 33 point-mutations and 27 complex-mutations. Fifty-three previously unreported unique *NOTCH1 *mutations were found in our series of Brazilian patients. In the *NOTCH1 *literature reports, the description of new mutations is a relatively frequent event [3,25,28-30]. To exclude false-positive results leading to the large number of new mutations, direct sequencing analyses were performed in different laboratory conditions and the same mutations were amplified with concordant results proving that contamination was unlikely. Moreover, *NOTCH1 *mutation profile concerning the domain affected (HD, PEST, or both) and type of mutations (point or complex) agreed with previously reported molecular analysis [3,10].

Associations between *NOTCH1 *mutations and outcome have been inconsistent in different series of cases [7]. This discrepancy may be due to differences in the therapeutic protocols for each series. We have not found significance on prognosis for *NOTCH1 *status (mutated vs. WT) in our T-ALL cases, and this finding is in accordance to major T-ALL studies [9,25,28]. Additionally we found that complex mutations were associated with a longer survival time than point mutations (*p *= 0.031), although the impact of mutation type did not stand as a prognostic factor for EFS. We believe that this lack of significance in the EFS analysis could be due to the small number of patients (n = 38) with EFS and *NOTCH1 *type of mutations available data. Then, we indicate the need to replicate this finding in an independent group of patients. Given the distinct functional effects of different mutations in *NOTCH1 *domains [7,9], our results raises the issue of the potential differential role of *NOTCH1 *mutation in the course of disease. Although no functional study has addressed this issue, we speculate that complex mutations would alter the Notch1 protein structure more drastically than point mutations, so blast cells would be more vulnerable to therapy responses. It is also possible that, as complex mutations were seen associated with high WBC, cases with complex mutations their presence lead to an initial more aggressive treatment, and hence to a sustained survival.

Key pathways related to Notch1 include the Ras/MAP Kinase and PI3-kinase/Akt. For the Ras/MAP Kinase pathway, since the majority of the studies evaluated the impact of *KRAS *mutations in mouse models with T-cell neoplasia [31,32], our *KRAS *data provides additional information in the evaluation of frequency and prognostic impact of these mutations in the largest described series of T-ALL patients. We found it in 9.5% of cases, without any impact on outcome. For the *crosstalk *between PI3-kinase/Akt and Notch1 pathways is described that Notch1 signalling via *Hes1 *down-regulates *PTEN*, an important negative regulator of PI3-kinase/Akt signalling [33]. Even with clear functional interaction between *NOTCH1 *and *PTEN *genes, no association for mutational status of these genes was found. Regarding the survival analysis, *PTEN *mutations displayed no statistically significance impact in OS or EFS (Table 3). Our results are in agreement with those of Gutierrez *et al*. [34], but are discordant with previous Brazilian results [24], which found a negative prognostic impact of *PTEN *mutations in T-ALLs. This variance may be due to the different classification of mutations (biallelic vs. monoallelic vs. wild-type) performed by Jotta *et al*. [24], indicating that the role of *PTEN *mutations as prognostic markers in T-ALL patients is not yet fully defined.

Mutations affecting *FBXW7 *in leukemic cells mediate Notch1 pathway activation [35]. Since *NOTCH1 *and *FBXW7 *mutations lead to the same effect, the hypothesis that T-ALL with *FBXW7 *mutations would be clinically similar to those with activating *NOTCH1 *mutations is reasonable [20]. Based on this idea, we performed a joint mutational analysis for these genes and our data of *NOTCH1-FBXW7 *mutation rate was 56.4% of T-ALLs, which is in line with previous reports on childhood T-ALL [35,36]. Concerning survival analysis, *NOTCH1-FBXW7 *mutational status showed no statistically significant impact on OS and EFS analysis. This absence of significance on OS and EFS analysis is concordant with other previously described results in representative series of paediatric T-ALL [19,20,37].

We have previously shown that *SIL-TAL1 *was an age-dependent prognostic factor, when rare cases of T-ALL in children with less than 1 year-old were included [6]. However, the present analysis excluding such young cases demonstrates that *SIL-TAL1 *is an independent adverse prognostic factor in older children as well.

## Conclusion

In our series the status of *NOTCH1 *mutations alone or in combination with *FBXW7 *did not impacted in T-ALL prognosis. Nevertheless, *NOTCH1 *complex mutations were associated with a longer T-ALL overall survival, and *SIL-TAL1 *was validated as a marker of worse prognosis. These data should be tested in other series of cases to confirm the relevance of such analyses.

## Competing interests

The authors declare that they have no competing interests.

## Authors' contributions

MBM designed, performed and wrote the paper. MBM, TCB and AS analysed the *NOTCH1 *and *FBXW7 *sequencing data. RH did all the statistical analyses. PYJ, JAY, RH and JLW helped with technical approaches, results discussions and data analyzes. MSPO designed and supervised the study and wrote the manuscript. All authors approved the manuscript descriptions.

## Pre-publication history

The pre-publication history for this paper can be accessed here:

http://www.biomedcentral.com/1471-2407/12/9/prepub

## Supplementary Material

Additional file 1 **Table S1**. Detailed descriptions of all NOTCH1 mutations sequences according to domain involved. Click here for file

Additional file 2 **Figure S1**. Kaplan-Meier overall survival curves for T-ALL patients. (A) Overall survival (OS) according to the presence of KRAS mutations. One hundred and five cases were included in this analysis, 10 with KRAS mutated and 95 WT. (B) OS according to the presence of PTEN mutations. In the PTEN OS analysis were included 96 patients, being 9 with mutations and 87 WT. (C) OS according to the expression of the TLX3 gene. For the construction of this OS curve, 119T-ALL cases were analyzed, 10 TLX3+ and 109 TLX3-(D) OS of patients treated with GBTLI (Brazilian Group for Treatment of Childhood Leukaemia) therapeutic protocol compared with ALL-BFM (Berlin-Frankfurt-Munster) protocols backbone strategies. Symbols represent censored cases. All 138 cases were included for Treatment Protocol OS analysis, 101 treated according GBTLI and 37 according BFM. Click here for file
