# Supplementary material for: Impact of complex NOTCH1 mutations on survival in paediatric T-cell leukaemia
Source: BMC Cancer. 2012 Jan 6;12:9. doi: 10.1186/1471-2407-12-9 (PMC3305583; doi:10.1186/1471-2407-12-9)
Supplement: Additional file 1 — Table S1. Detailed descriptions of all NOTCH1 mutations sequences according to domain involved. [file 1471-2407-12-9-S1.DOC]

| **Additional Table 1 - Detailed descriptions of all *NOTCH1* mutations sequences according to domain involved.** | | | |
| --- | --- | --- | --- |
| Cases ID | Mutations Status | HD Domain Alterations^a^ | |
| 1, 11, 14, 16, 17, 18, 19, 21, 22, 23, 26, 29, 30, 31, 38, 42, 43, 45, 46, 53, 60, 63,  64, 65, 68, 72, 73, 75, 77, 78, 80 | R | c.5094C>T (SNP - rs10521) | p.D1698D |
| 2 | N | c.4649A>C | p.N1550T |
| 3 | N | c.4776_ 4777insTAGGGC | p.F1592_L1593insRA |
| 4, 79 | N | c.4918_4939delGCACCTGACGCCCTGCTGGGCC homo/hemi | p. A1640R ; p.P1641X |
| 6, 9 | R | c.4757T>C | p.L1586P |
| 7 | N | g.42407 C>T (intron) | - |
| 7, 27 | N | c.5123C>A | p.A1708N |
| 8, 44, 55 | R | c.5011G>A (SNP - rs115563691) | p.V1671I |
| 7, 11, 16, 20, 20, 21 | N | c.5025C>T | p.I1675I |
| 11 | N | c.5070_5071insATGTCGGGCATGATTCAATTGAGGTA | p.1691_1692insCRAX |
| 11, 16, 68 | R | c.5033T>C | p.L1678P |
| 17 | N | c.4901C>T | p.A1634V |
| 17 | N | c.4921C>T | p.P1641S |
| 17 | N | c.4939C>T | p.Q1647X |
| 17 | N | c.4968C>T | p.G1656G |
| 17 | N | c.4987C>T | p.R1663W |
| 17 | N | c. 4996C>T | p.L1666L |
| 20 | R | c.4729G>A | p.V1577M |
| 20, 67 | N | c.5026_5027insTCGTCGACCGGAGATTGACAAC | p.1676_1677insSSTGDX |
| 21 | R | c.4811T>G | p.V1604G |
| 22 | N | c.5096_5097insCAGTGCTTCCAGAGTGC | p.1699_1700insAVLPECWPHSWERSPRWAASTSPTRSRPCR |
| 23, 25 | N | c.4833C>T | p.H1611H |
| 25 | N | c.5028_5030delCTA | p.1677delY |
| 26 | R | c.4754T>A | p.L1585Q |
| 31 | R | c.4793G>C | p.R1598P |
| 31, 55 | N | c.4905C>T | p.A1635A |
| 31 | R | c.4927G>A | p.A1643T |
| 35 | N | c.4670G>T | p.W1556L |
| 36 | R | c.4781T>C | p.L1593P |
| 40 | R | c.4799T>C | p.L1600P |
| 42, 54, 61 | R | c.4735_4737delGTG | p.1578delV |
| 45 | R | c.4721T>C | p.L1574P |
| 46 | N | c.4987_4989delCGG | p.1663delR |
| 47 | N | c.4906_4941delGAGGGCTGGGCCGCACCTGACGCCCTGCTGGGCCAG | p.1636_1647delEGWAAPDALLGQ |
| 47 | N | c.4971C>T | p.S1657S |
| 48 | R | c.4948G>A | p.A1650T |
| 48 | N | c.4918G>A | p.A1640T |
| 51 | N | c.4897C>T homo | p.R1633C |
| 51, 70 | N | c.4915G>A homo | p.A1639T |
| 52, 55 | N | c.4956G>A | p.L1652L |
| 55 | R | c.4754T>C | p.L1585P |
| 56 | N | c.4984C>T | p.R1662W |
| 58, 66 | N | c.4903G>A homo | p.A1635T |
| 58 | N | c.4953G>A homo | p.S1651S |
| 62 | R | c.4775T>C | p.F1592S |
| 62 | N | c.4939C>T homo | p.Q1647X |
| 62 | R | c.4976G>A homo | p.G1659D |
| 64 | R | c.4818_4819delCAinsGGGGTCAC | p.F1606L; p.1606_1607delKinsGSQ |
| 65 | N | c.4906G>A | p.E1636K |
| 65 | N | c.4933C>T | p.L1645L |
| 66 | N | c.4979G>A | p.G1660E |
| 70 | N | c. 4932G>A | p.L1644L |
| 71 | N | c.4866C>T | p.R1622R |
| 71 | N | c.4873_4911delGAGCTGCGCAAGCACCCCATCAAGCGTGCCGCCGAGGGC | p.1625_1637delELRKHPIKRAAEG |
| 74 | R | c.4790G>T | p.S1597I |
| 76 | N | c.5015G>A | p. R1672H |
| 79 | N | c.4899T>C | p.R1633R |
| Cases ID | Mutations Status | PEST/TAD Domains Alterations^a^ | |
| 4 | N | c.7254delC | p.G2420A; V2421X |
| 4 | N | c.7258G>C | p.G2420R |
| 5, 15 | R | c.6991G>A (SNP - rs111309246) | p.A2331T |
| 8, 55 | R | c.7515T>G (SNP - rs34152221) | p.P2505P |
| 9 | N | c.7171_7172del CinsAGTGCCAAA | p.2391_2392insSAKRCSSRTCSQQTSSSSKACSRHHHHHSRTLA |
| 10, 13, 23, 26, 41, 63, 64, 73, 76 | R | c.6853G>A (SNP - rs61751489) | p.V2285I |
| 11 | R | c.7005_7006insT | p.2336_2337insEHTGPLPAAWHGRPAAQX |
| 12 | N | c.7648A>G | p.I2550V |
| 12 | N | c.7177_7178insAG | p.2393_2394insSSSRTCSQQTSSSSKACSRHHHHHSRTLAX |
| 15 | R | c.6870C>T | p.S2290S |
| 15, 24, 32, 37, 48, 69 | R | c.7233A>G (SNP - rs11574911) | p.P2411P |
| 19 | N | c.7485_7486insGGAATGT | p.2495_2496insEECQHPQPPATGAX |
| 20 | N | c.7230G>T | p.P2410P |
| 24, 28, 50, 69 | R | c.7674G>A (SNP - rs114065541) | 3'UTR |
| 29 | N | c.7328_7329insGG | p.2443_2444insGCSHWAPAAWRCTLFCPRRAPPCPRRCHPRWSHP |
| 33 | R | c.7541_7542delCT | p.2514_2515RVPX |
| 34 | R | c.7289G>C (SNP - rs36049318) | p.G2430A |
| 39 | N | c.7311_7313delGCCinsAT | p.P2438X |
| 41 | N | c.7413_7414insAA | p.2472_2473insSWSHPX |
| 47 | N | c.7648A>G | p.I2550V |
| 48 | N | c.6921A>G | p.Q2307Q |
| 49 | R | c.6985_6986insG | p.2329_2330ins ECGTRPPEHTGPLPAAWHGRPAAQX |
| 53 | R | c.7609G>A | p.V2536I |
| 57 | N | c.7355_7356insAG | p.2452_2453ERCTLFCPRRAPPCPRRCHPRWSHPX |
| 59 | N | c.7028A>T | p.Q2343L |
| 71 | R | c.7479T>G | p.P2493P |
| 72 | N | c.6856G>A | p.V2285V |

^a^ Nucleotide and amino acids numbers are according to the GenBank accession numbers NM_017617.3 and NP_060087.3, respectively. R, previously reported; N, new mutation, previously undescribed. SNPs were determined using NCBI SNP database and 1000 genomes data. Among all new mutations there were no SNPs, since we have checked and excluded any SNP possibility through NCBI SNP database and 1000 genomes data.
