# Supplementary figures and images for: Impact of complex NOTCH1 mutations on survival in paediatric T-cell leukaemia
Source: BMC Cancer. 2012 Jan 6;12:9. doi: 10.1186/1471-2407-12-9 (PMC3305583; doi:10.1186/1471-2407-12-9)

## Slide 1
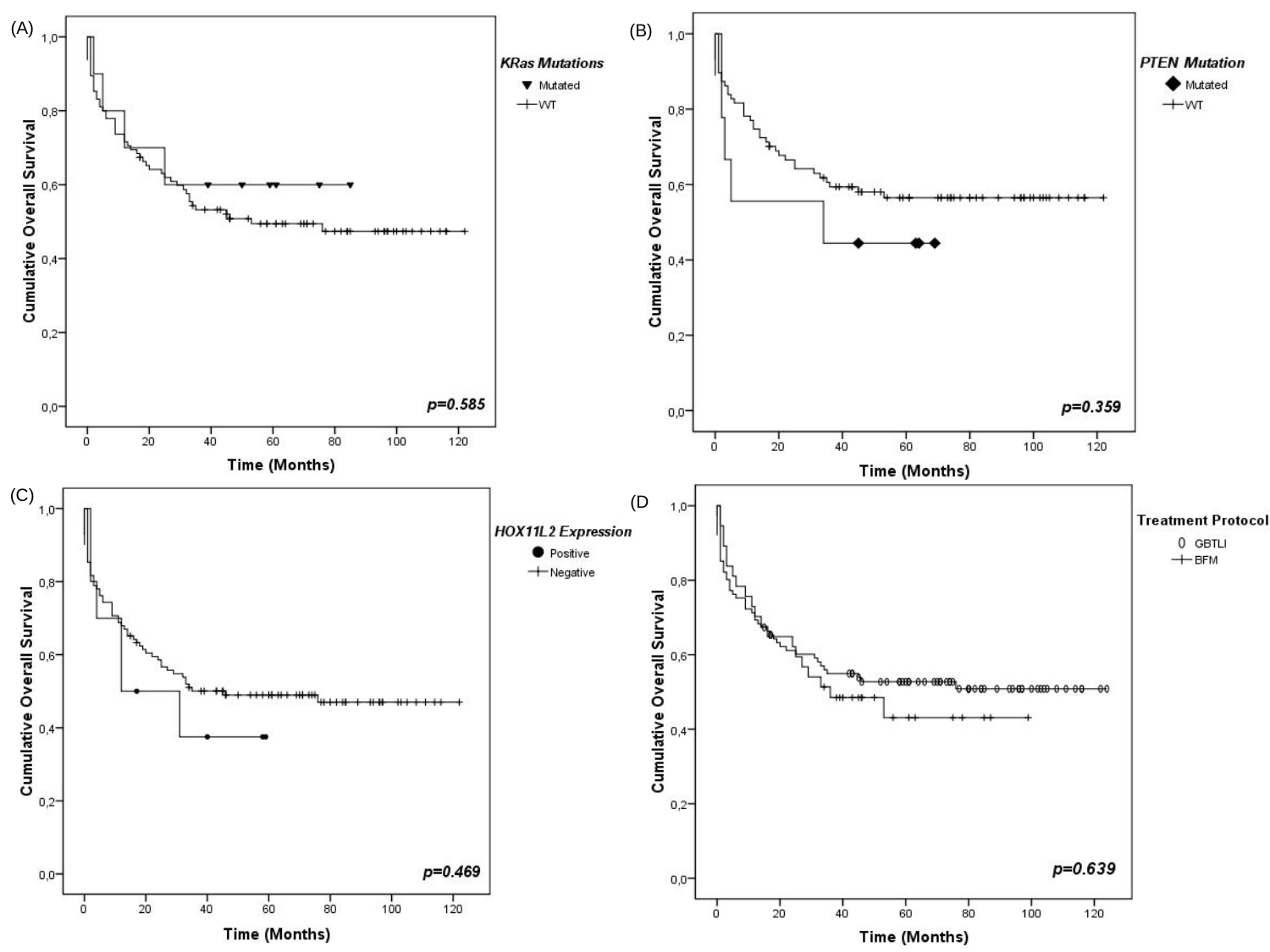

(A)
(B)
(C)
(D)

Supplement: Additional file 2 — Figure S1. Kaplan-Meier overall survival curves for T-ALL patients. (A) Overall survival (OS) according to the presence of KRAS mutations. One hundred and five cases were included in this analysis, 10 with KRAS mutated and 95 WT. (B) OS according to the presence of PTEN mutations. In the PTEN OS analysis were included 96 patients, being 9 with mutations and 87 WT. (C) OS according to the expression of the TLX3 gene. For the construction of this OS curve, 119T-ALL cases were analyzed, 10 TLX3+ and 109 TLX3-(D) OS of patients treated with GBTLI (Brazilian Group for Treatment of Childhood Leukaemia) therapeutic protocol compared with ALL-BFM (Berlin-Frankfurt-Munster) protocols backbone strategies. Symbols represent censored cases. All 138 cases were included for Treatment Protocol OS analysis, 101 treated according GBTLI and 37 according BFM. [file 1471-2407-12-9-S2.PPT]
